# 5-(2-Hy­droxy­phen­yl)-3-methyl-4,5-di­hydro-1*H*-pyrazole-1-carbaldehyde

**DOI:** 10.1107/S1600536810031600

**Published:** 2010-08-18

**Authors:** Ping Cui, Xin-Long Li

**Affiliations:** a School of Chemistry and Chemical Engineering, Anhui University of Technology, Maanshan 243002, People’s Republic of China

## Abstract

In the title compound, C_11_H_12_N_2_O_2_, the dihydro­pyrazole and benzene rings are oriented at a dihedral angle of 68.35 (5)°. The dihydro­pyrazole ring is planar, with a mean deviation from the mean plane of 0.0409 Å. The crystal structure is stabilized by O—H⋯O and C—H⋯O hydrogen bonds.

## Related literature

For the anti­bacterial bioactivity of pyrazole derivatives, see: Bekhita & Abdel-Aziem (2004 ▶); Tanitame *et al.* (2004*a* ▶,*b* ▶). For the biological properties of dihydro­pyrazole derivatives, see: Dmytro *et al.* (2009 ▶); Need *et al.* (2006 ▶).
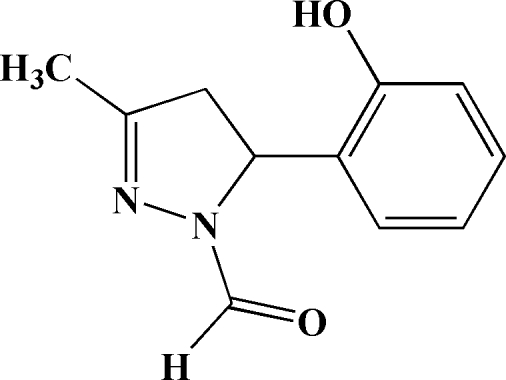


## Experimental

### 

#### Crystal data

C_11_H_12_N_2_O_2_
*M* *_r_* = 204.23
Monoclinic, 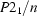

*a* = 7.3835 (15) Å
*b* = 13.454 (3) Å
*c* = 10.507 (2) Å
β = 106.46 (3)°
*V* = 1001.0 (3) Å^3^
*Z* = 4
Mo *K*α radiation
μ = 0.10 mm^−1^
*T* = 293 K
0.21 × 0.16 × 0.11 mm

#### Data collection

Bruker SMART APEX CCD area-detector diffractometer
Absorption correction: multi-scan (*SADABS*; Bruker, 2001 ▶) *T*_min_ = 0.980, *T*_max_ = 0.990
5606 measured reflections
1957 independent reflections
1579 reflections with *I* > 2σ(*I*)
*R*_int_ = 0.019

#### Refinement

*R*[*F* ^2^ > 2σ(*F* ^2^)] = 0.033
*wR*(*F* ^2^) = 0.086
*S* = 1.08
1957 reflections
138 parameters
H-atom parameters constrained
Δρ_max_ = 0.20 e Å^−3^
Δρ_min_ = −0.18 e Å^−3^

### 

Data collection: *SMART* (Bruker, 2001 ▶); cell refinement: *SAINT* (Bruker, 2001 ▶); data reduction: *SAINT*; program(s) used to solve structure: *SHELXS97* (Sheldrick, 2008 ▶); program(s) used to refine structure: *SHELXL97* (Sheldrick, 2008 ▶); molecular graphics: *SHELXTL* (Sheldrick, 2008 ▶); software used to prepare material for publication: *SHELXL97*.

## Supplementary Material

Crystal structure: contains datablocks global, I. DOI: 10.1107/S1600536810031600/ez2216sup1.cif

Structure factors: contains datablocks I. DOI: 10.1107/S1600536810031600/ez2216Isup2.hkl

Additional supplementary materials:  crystallographic information; 3D view; checkCIF report

## Figures and Tables

**Table 1:** Hydrogen-bond geometry (Å, °)

*D*—H⋯*A*	*D*—H	H⋯*A*	*D*⋯*A*	*D*—H⋯*A*
O2—H2⋯O1^i^	0.82	1.88	2.6954 (14)	175
C4—H4*B*⋯O1^ii^	0.97	2.48	3.4438 (16)	170

Symmetry codes: (i) 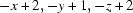; (ii) 
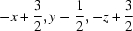
.

**Fig. 1.**
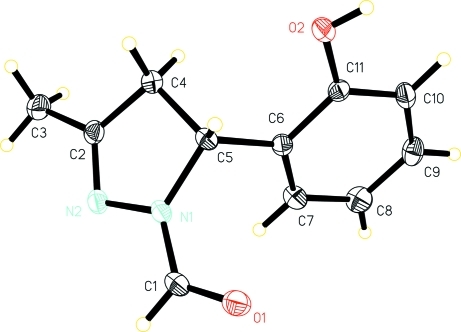
The crystal structure of (I), showing the atom numbering scheme and 35% probability displacement ellipsoids (arbitrary spheres for the H atoms).

**Fig. 2.**
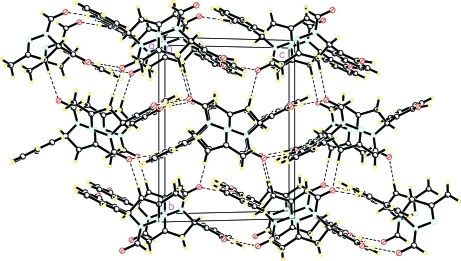
The crystal packing of (I), viewed along the a axis. Hydrogen bonds are shown as dashed lines.

## supplementary crystallographic information

### Comment

There has been much research interest in pyrazole derivatives due to their antibacterial bioactivities (Bekhita *et al.*, 2004; Tanitame *et al.*, 2004*a*; Tanitame *et al.*, 2004*b*). Dihydropyrazole-based derivatives have shown several biological activities as CB1 antagonists and tumor necrosis inhibitors (Dmytro *et al.*, 2009; Need *et al.*, 2006). In this paper, we report the synthesis and crystal structure of 5-(2-hydroxyphenyl)-3-methyl-4,5-dihydropyrazole-1-carbaldehyde (I).

The title compound crystallizes in the centrosymmetric space group *P*2_1_/*n*. As shown in Fig. 1, the C—N single and double bond lengths are both in the normal ranges [C5—N1 single bond is 1.4826 (15) Å, C12═N2 double bond is 1.2797 (17) Å]. The bond lengths of C3—C12 and C3—C5 are 1.5002 (17) and 1.5421 (18) Å, respectively, which indicate that they are both single bonds. The dihedral angle between the dihydropyrazole and benzene rings is 68.35 (5) °. The dihydropyrazole ring adopts a planar conformation, with a mean deviation from the mean plane of 0.0409 Å. The intermolecular O2—H2···O1 and C4—H4B···O1 hydrogen bonds connect the molecules to form a three-dimensional network (Fig. 2).

### Experimental

To a solution of 4-(2-hydroxyphenyl)but-3-en-2-one (0.81 g, 5 mmol) in formic acid (10 ml) was added hydrazine monohydrate (1.25 ml, 25 mmol) and the reaction mixture was refluxed for 2 h. The solvent was evaporated and cold water (30 ml) was added to the oily residue. The resultant precipitate was filtered, recrystallized from ethanol, and colorless single crystals were obtained after 1 day. Mp 155–156 °C. Analysis found: C, 64.5; H, 6.1; N, 14.0%; calculated for C_11_H_12_N_2_O_2_: C, 64.7; H, 5.9; N, 13.7%. ^1^H NMR (300 MHz, CDCl_3_, δ, p.p.m.): 2.19 (s, 3H, –Me), 3.10 (dd, J = 18.7 and 3.7 Hz, 1H, pyrazole), 3.39 (dd, J = 11.2 and 18.6 Hz, 1H, pyrazole), 5.67 (dd, J = 11.6 and 3.5 Hz, 1H, pyrazole), 6.89–7.26 (m, 5H, ArH and –OH), 8.63 (s, 1H, –COH).

### Refinement

All the H atoms were placed in idealized positions (C—H = 0.93–0.97, O—H = 0.82 Å) and refined as riding with *U*_iso_(H) = 1.2*U*_eq_(C) or 1.5*U*_eq_(C) for methyl H atoms and *U*_iso_(H) = 1.5*U*_eq_(O).

### Crystal data

**Table:**

C_11_H_12_N_2_O_2_	*F*(000) = 432
*M**_r_* = 204.23	*D*_x_ = 1.355 Mg m^−^^3^
Monoclinic, *P*2_1_/*n*	Mo *K*α radiation, λ = 0.71073 Å
*a* = 7.3835 (15) Å	Cell parameters from 972 reflections
*b* = 13.454 (3) Å	θ = 3.5–24.7°
*c* = 10.507 (2) Å	µ = 0.10 mm^−^^1^
β = 106.46 (3)°	*T* = 293 K
*V* = 1001.0 (3) Å^3^	Block, colorless
*Z* = 4	0.21 × 0.16 × 0.11 mm

### Data collection

**Table:**

Bruker Smart APEX CCD area-detector diffractometer	1957 independent reflections
Radiation source: fine-focus sealed tube	1579 reflections with *I* > 2σ(*I*)
graphite	*R*_int_ = 0.019
phi and ω scans	θ_max_ = 26.0°, θ_min_ = 3.0°
Absorption correction: multi-scan (*SADABS*; Bruker, 2001)	*h* = −9→8
*T*_min_ = 0.980, *T*_max_ = 0.990	*k* = −16→13
5606 measured reflections	*l* = −12→12

### Refinement

**Table:**

Refinement on *F*^2^	Primary atom site location: structure-invariant direct methods
Least-squares matrix: full	Secondary atom site location: difference Fourier map
*R*[*F*^2^ > 2σ(*F*^2^)] = 0.033	Hydrogen site location: inferred from neighbouring sites
*wR*(*F*^2^) = 0.086	H-atom parameters constrained
*S* = 1.08	*w* = 1/[σ^2^(*F*_o_^2^) + (0.0484*P*)^2^ + 0.0583*P*] where *P* = (*F*_o_^2^ + 2*F*_c_^2^)/3
1957 reflections	(Δ/σ)_max_ < 0.001
138 parameters	Δρ_max_ = 0.20 e Å^−^^3^
0 restraints	Δρ_min_ = −0.18 e Å^−^^3^

### Special details

**Table:**

Geometry. All e.s.d.'s (except the e.s.d. in the dihedral angle between two l.s. planes) are estimated using the full covariance matrix. The cell e.s.d.'s are taken into account individually in the estimation of e.s.d.'s in distances, angles and torsion angles; correlations between e.s.d.'s in cell parameters are only used when they are defined by crystal symmetry. An approximate (isotropic) treatment of cell e.s.d.'s is used for estimating e.s.d.'s involving l.s. planes.
Refinement. Refinement of *F*^2^ against ALL reflections. The weighted *R*-factor *wR* and goodness of fit *S* are based on *F*^2^, conventional *R*-factors *R* are based on *F*, with *F* set to zero for negative *F*^2^. The threshold expression of *F*^2^ > σ(*F*^2^) is used only for calculating *R*-factors(gt) *etc*. and is not relevant to the choice of reflections for refinement. *R*-factors based on *F*^2^ are statistically about twice as large as those based on *F*, and *R*- factors based on ALL data will be even larger.

### Fractional atomic coordinates and isotropic or equivalent isotropic displacement parameters (Å^2^)

**Table:**

	*x*	*y*	*z*	*U*_iso_*/*U*_eq_	
C1	0.71057 (17)	0.62387 (9)	0.66395 (12)	0.0271 (3)	
H1	0.6378	0.6593	0.5914	0.032*	
C2	0.81434 (16)	0.41527 (9)	0.51386 (12)	0.0253 (3)	
C3	0.7931 (2)	0.35621 (10)	0.39114 (13)	0.0350 (3)	
H3A	0.7264	0.2958	0.3963	0.052*	
H3B	0.9157	0.3406	0.3820	0.052*	
H3C	0.7235	0.3941	0.3157	0.052*	
C4	0.93391 (17)	0.38472 (9)	0.64868 (12)	0.0260 (3)	
H4A	1.0670	0.3859	0.6534	0.031*	
H4B	0.9004	0.3187	0.6715	0.031*	
C5	0.88677 (16)	0.46406 (8)	0.74008 (11)	0.0225 (3)	
H5	1.0031	0.4963	0.7925	0.027*	
C6	0.77821 (16)	0.42376 (8)	0.83124 (11)	0.0215 (3)	
C7	0.58407 (17)	0.42893 (9)	0.80332 (12)	0.0284 (3)	
H7	0.5137	0.4564	0.7234	0.034*	
C8	0.49282 (18)	0.39382 (10)	0.89242 (14)	0.0341 (3)	
H8	0.3621	0.3985	0.8730	0.041*	
C9	0.59650 (18)	0.35179 (10)	1.01025 (13)	0.0320 (3)	
H9	0.5357	0.3294	1.0712	0.038*	
C10	0.78979 (18)	0.34274 (9)	1.03825 (12)	0.0276 (3)	
H10	0.8587	0.3126	1.1167	0.033*	
C11	0.88131 (16)	0.37877 (8)	0.94901 (11)	0.0221 (3)	
N1	0.77351 (14)	0.53492 (7)	0.64102 (9)	0.0237 (2)	
N2	0.72976 (14)	0.49870 (8)	0.51056 (9)	0.0259 (3)	
O1	0.74131 (13)	0.66270 (6)	0.77367 (9)	0.0335 (2)	
O2	1.07147 (12)	0.37142 (7)	0.97002 (8)	0.0306 (2)	
H2	1.1227	0.3625	1.0493	0.046*	

### Atomic displacement parameters (Å^2^)

**Table:**

	*U*^11^	*U*^22^	*U*^33^	*U*^12^	*U*^13^	*U*^23^
C1	0.0288 (6)	0.0248 (7)	0.0264 (7)	0.0022 (5)	0.0059 (5)	0.0068 (5)
C2	0.0238 (6)	0.0293 (7)	0.0251 (6)	−0.0015 (5)	0.0107 (5)	0.0023 (5)
C3	0.0377 (7)	0.0404 (8)	0.0272 (7)	0.0047 (6)	0.0099 (6)	−0.0024 (6)
C4	0.0282 (6)	0.0268 (7)	0.0248 (6)	0.0030 (5)	0.0104 (5)	0.0037 (5)
C5	0.0231 (6)	0.0216 (6)	0.0219 (6)	0.0008 (5)	0.0049 (5)	0.0044 (5)
C6	0.0266 (6)	0.0175 (6)	0.0208 (6)	−0.0009 (5)	0.0073 (5)	−0.0007 (4)
C7	0.0262 (6)	0.0293 (7)	0.0282 (7)	−0.0012 (5)	0.0051 (5)	0.0045 (5)
C8	0.0249 (6)	0.0382 (8)	0.0407 (8)	−0.0045 (5)	0.0117 (6)	0.0015 (6)
C9	0.0383 (7)	0.0324 (7)	0.0306 (7)	−0.0097 (6)	0.0183 (6)	−0.0016 (5)
C10	0.0383 (7)	0.0248 (7)	0.0203 (6)	−0.0020 (5)	0.0092 (5)	0.0012 (5)
C11	0.0264 (6)	0.0184 (6)	0.0218 (6)	0.0009 (5)	0.0074 (5)	−0.0028 (5)
N1	0.0286 (5)	0.0233 (5)	0.0188 (5)	0.0018 (4)	0.0059 (4)	0.0037 (4)
N2	0.0275 (5)	0.0306 (6)	0.0200 (5)	0.0001 (4)	0.0072 (4)	0.0026 (4)
O1	0.0418 (5)	0.0275 (5)	0.0279 (5)	0.0067 (4)	0.0047 (4)	−0.0002 (4)
O2	0.0267 (5)	0.0416 (6)	0.0228 (4)	0.0073 (4)	0.0059 (3)	0.0066 (4)

### Geometric parameters (Å, °)

**Table:**

C1—O1	1.2269 (15)	C5—H5	0.9800
C1—N1	1.3303 (16)	C6—C7	1.3811 (17)
C1—H1	0.9300	C6—C11	1.3940 (16)
C2—N2	1.2802 (16)	C7—C8	1.3824 (19)
C2—C3	1.4846 (18)	C7—H7	0.9300
C2—C4	1.4988 (17)	C8—C9	1.3790 (19)
C3—H3A	0.9600	C8—H8	0.9300
C3—H3B	0.9600	C9—C10	1.3785 (18)
C3—H3C	0.9600	C9—H9	0.9300
C4—C5	1.5403 (17)	C10—C11	1.3892 (18)
C4—H4A	0.9700	C10—H10	0.9300
C4—H4B	0.9700	C11—O2	1.3617 (15)
C5—N1	1.4828 (14)	N1—N2	1.4034 (14)
C5—C6	1.5129 (17)	O2—H2	0.8200
			
O1—C1—N1	124.90 (11)	C7—C6—C11	118.90 (12)
O1—C1—H1	117.5	C7—C6—C5	123.48 (11)
N1—C1—H1	117.5	C11—C6—C5	117.62 (11)
N2—C2—C3	121.01 (11)	C6—C7—C8	120.97 (12)
N2—C2—C4	114.73 (11)	C6—C7—H7	119.5
C3—C2—C4	124.26 (11)	C8—C7—H7	119.5
C2—C3—H3A	109.5	C9—C8—C7	119.67 (12)
C2—C3—H3B	109.5	C9—C8—H8	120.2
H3A—C3—H3B	109.5	C7—C8—H8	120.2
C2—C3—H3C	109.5	C10—C9—C8	120.38 (13)
H3A—C3—H3C	109.5	C10—C9—H9	119.8
H3B—C3—H3C	109.5	C8—C9—H9	119.8
C2—C4—C5	102.86 (9)	C9—C10—C11	119.81 (12)
C2—C4—H4A	111.2	C9—C10—H10	120.1
C5—C4—H4A	111.2	C11—C10—H10	120.1
C2—C4—H4B	111.2	O2—C11—C10	122.79 (11)
C5—C4—H4B	111.2	O2—C11—C6	117.01 (11)
H4A—C4—H4B	109.1	C10—C11—C6	120.20 (11)
N1—C5—C6	112.35 (10)	C1—N1—N2	119.59 (9)
N1—C5—C4	100.95 (9)	C1—N1—C5	127.43 (10)
C6—C5—C4	113.59 (10)	N2—N1—C5	112.98 (9)
N1—C5—H5	109.9	C2—N2—N1	107.61 (9)
C6—C5—H5	109.9	C11—O2—H2	109.5
C4—C5—H5	109.9		
			
N2—C2—C4—C5	7.20 (14)	C7—C6—C11—O2	177.26 (11)
C3—C2—C4—C5	−173.35 (11)	C5—C6—C11—O2	−2.42 (15)
C2—C4—C5—N1	−8.69 (12)	C7—C6—C11—C10	−2.02 (17)
C2—C4—C5—C6	111.79 (11)	C5—C6—C11—C10	178.30 (10)
N1—C5—C6—C7	17.36 (16)	O1—C1—N1—N2	−179.59 (11)
C4—C5—C6—C7	−96.46 (14)	O1—C1—N1—C5	1.7 (2)
N1—C5—C6—C11	−162.98 (10)	C6—C5—N1—C1	66.03 (15)
C4—C5—C6—C11	83.21 (13)	C4—C5—N1—C1	−172.61 (11)
C11—C6—C7—C8	2.53 (19)	C6—C5—N1—N2	−112.71 (11)
C5—C6—C7—C8	−177.80 (12)	C4—C5—N1—N2	8.64 (12)
C6—C7—C8—C9	−0.9 (2)	C3—C2—N2—N1	178.62 (11)
C7—C8—C9—C10	−1.4 (2)	C4—C2—N2—N1	−1.92 (14)
C8—C9—C10—C11	1.87 (19)	C1—N1—N2—C2	176.47 (11)
C9—C10—C11—O2	−179.39 (11)	C5—N1—N2—C2	−4.68 (13)
C9—C10—C11—C6	−0.16 (18)		

### Hydrogen-bond geometry (Å, °)

**Table:**

*D*—H···*A*	*D*—H	H···*A*	*D*···*A*	*D*—H···*A*
O2—H2···O1^i^	0.82	1.88	2.6954 (14)	175.
C4—H4B···O1^ii^	0.97	2.48	3.4438 (16)	170.

Symmetry codes: (i) −*x*+2, −*y*+1, −*z*+2; (ii) −*x*+3/2, *y*−1/2, −*z*+3/2.
